# Using 3D computer planning for complex reconstruction of mandibular defects

**DOI:** 10.1186/s41199-016-0019-4

**Published:** 2016-12-09

**Authors:** Diana N. Kirke, Randall P. Owen, Vincent Carrao, Brett A Miles, Jason I. Kass

**Affiliations:** 1 Department of Otolaryngology Head & Neck Surgery, Boston Medical Center, Boston, MA USA; 2 Departments of General Surgery, The Icahn School of Medicine at Mount Sinai, New York, NY USA; 3 Oral & Maxillofacial Surgery, The Icahn School of Medicine at Mount Sinai, New York, NY USA; 4 Otolaryngology Head & Neck Surgery, The Icahn School of Medicine at Mount Sinai, New York, NY USA; 5 Department of Surgery, VA Boston Healthcare System, Jamaica Plain, 150 South Huntington Avenue, Boston, MA 02130 USA

**Keywords:** 3D, Computer planning, Head and neck, Mandible, Osseous defects, Reconstruction, Virtual surgical planning

## Abstract

For complex reconstruction of osseous defects of the head and neck, three-dimensional (3D) computer planning has been available for over 20 years. However, despite its availability and recent refinements, it is a technology that has not been widely adopted. While 3D computer planning has been proposed to improve surgical precision, reduce operating time and enhance functional outcomes, the objective evidence supporting these claims is limited. Here we review the recent literature that supports the use of 3D computer planning for complex osseous defects of the mandible. We highlight a case example where 3D modeling played a critical role, particularly during the virtual surgical planning stage. Finally, we propose that routine post-operative 3D analysis become an essential element in determining operative success. Critical evaluation of outcomes will better define its use in complex reconstruction of osseous defects.

## Background

3D planning for reconstruction of osseous defects of the head and neck is a computer-based surgical planning system that has been present now for over 20 years [1, 2]. 3D planning can also be described as virtual surgical planning (VSP), computer-aided design (CAD) or computer assisted modeling (CAM), but all terms are synonymous with a concept that utilize preoperative virtual simulation and planning of the proposed osseous defect, rather than relying solely on traditional intraoperative manual assessment. Despite both the availability (in the USA) and ongoing refinement of virtual planning software systems, it is still not in routine use [3]. Additionally, this technology is not readily available internationally [4]. Applications for its use thus far, in the head and neck, have included complex craniofacial surgery, osseous reconstruction following resection of both benign and malignant tumors and osteoradionecrosis (ORN) [1, 2, 5–8]. The proposed benefits of 3D planning for reconstruction include improved surgical precision, reduced operating time and ultimately improved structural and functional reconstructive outcomes. In addition, the reduced operative times may ultimately result in reduced cost, despite the costs of additional technology [9–12]. The disadvantages include the potential for prolonged pre–operative planning, resulting in delays in care, and the inability to adjust to intraoperative changes (e.g. positive tumor margins). [10, 13, 14]. Currently, while there are proposed cases that are well suited for VSP, there are no definitive criteria [15]. This is likely, in part, a function of surgical volume, exposure/comfort with the technology, and an ability to justify the expense. This is particularly relevant since alternatives to VSP, including rulers or tongue depressors, are inexpensive, easily adjustable and versatile [16]. While conventional techniques can be applied for routine cases, VSP is particularly useful for the following: hemimandibulectomy, condyle reconstruction, large erosive lesions that preclude a pre-bent plate and symphyseal defects. It is our belief this technology will only be widely adopted once objective measures consistently demonstrate a benefit over traditional techniques.

### Case example

**Fig. 1:**
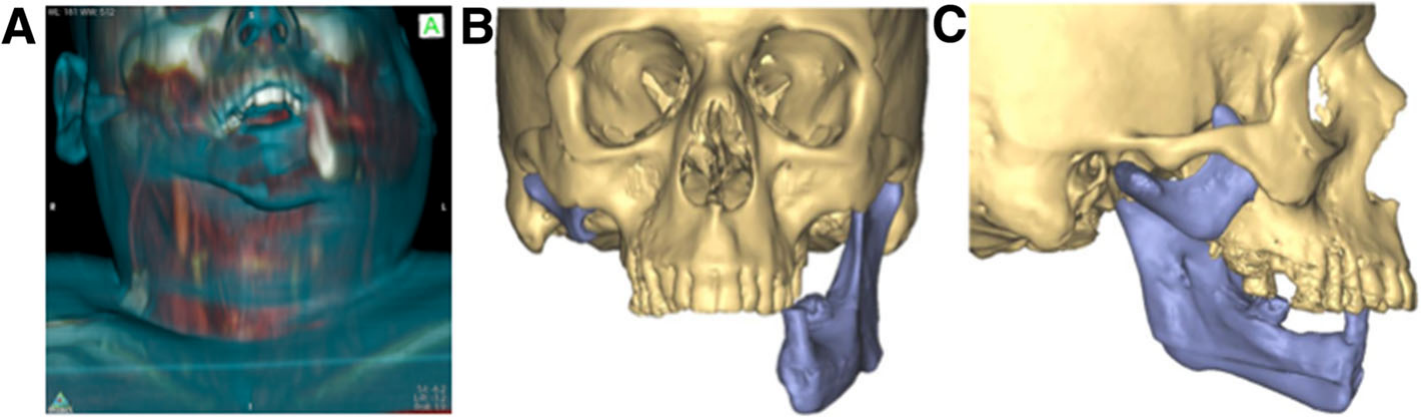
Pre-operative evaluation of a 43 year old status-post right hemi-mandibulectomy, chemotherapy and post-operative radiotherapy for Ewing Sarcoma. **a** Composite skin-bone CT projection showing soft tissue contour overlying right hemi-mandibular defect **b**. 3-D CT reconstruction showing remaining left hemi-mandible **c**. Remnant right condyle, neck and coronoid process

**Fig. 2:**
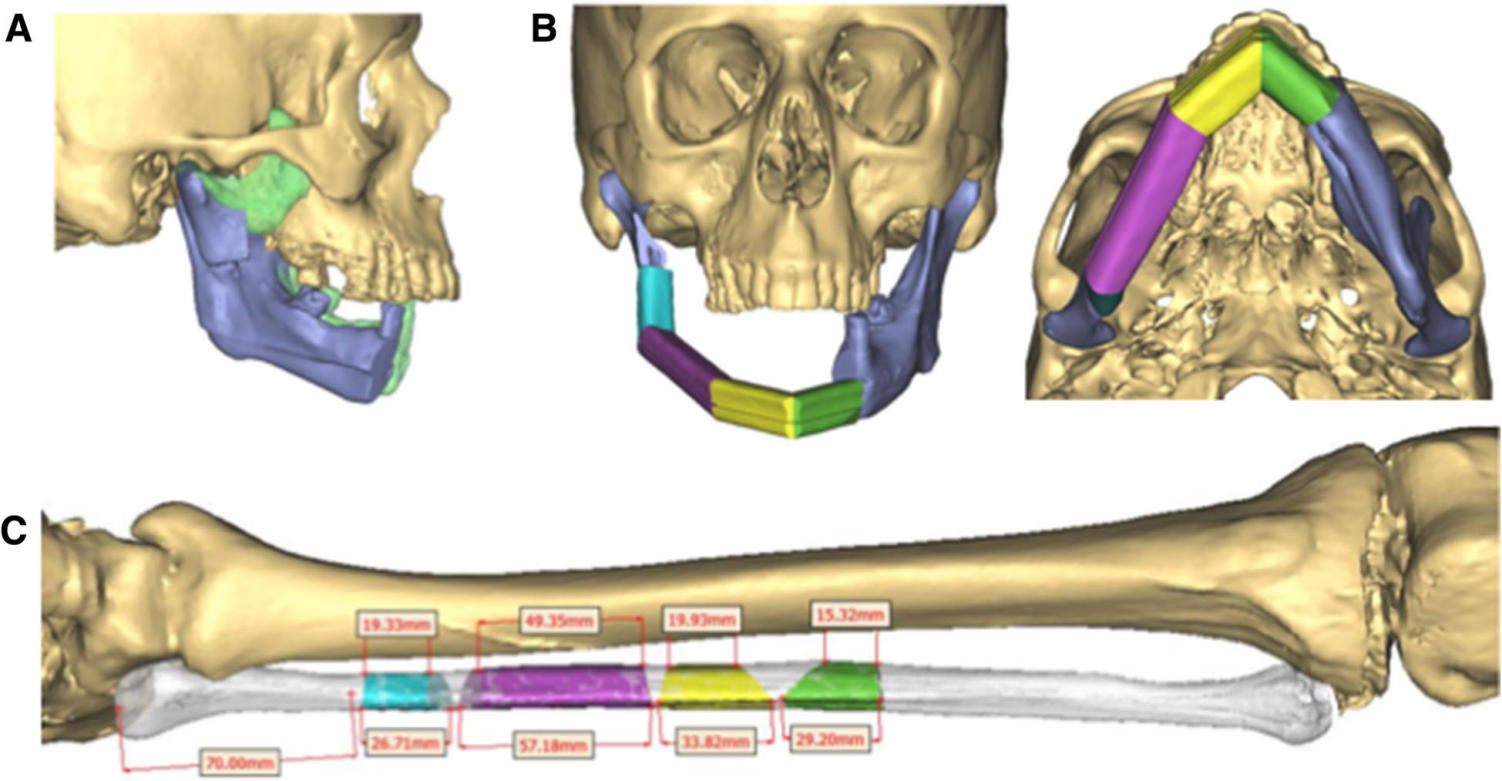
Planned reconstruction with 4 fibular segments. **a** 3-D CT reconstruction demonstrating resection of the remnant coronoid and repositioning of the right condyle. Original position shown in *green* with the planned reposition in *blue* **b**. Anterior and base views of the 4-segment reconstruction plan. **c** Placement of the proposed osteotomies on the virtual fibula

**Fig. 3:**
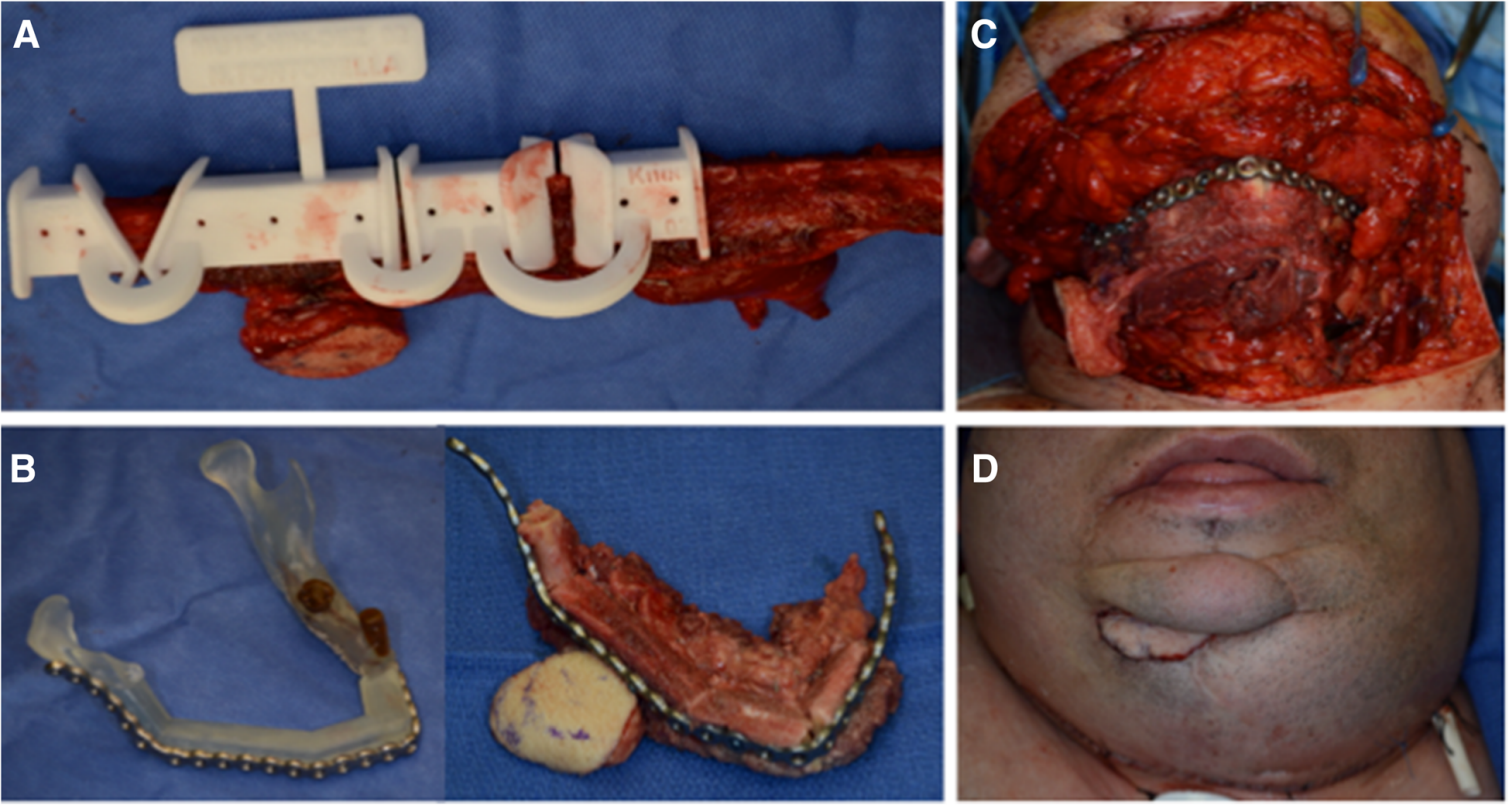
Intraoperative implementation of the virtual surgical plan. **a** Harvest of the left fibula with the cutting guide in place. **b** Pre-bent reconstruction bar contoured to pre-operative model (*left*) then attached to the fibular free flap following osteotomies (*right*). **c** Following plating of the fibular segments to the native mandible. **d** Post-operative result with a small external skin paddle

There are many excellent case reports [17–20] and case series [6–8, 13, 21–25] that describes the planning and implementation of computer-aided design (CAD) for complex head and neck reconstruction. Figures 1, 2 and 3 demonstrate a case that would otherwise be very challenging without computerized surgical planning and guides for execution. In this example we used VSP to (a) reposition a displaced remnant right mandibular condyle and (b) reconstruct a hemi-mandibulectomy defect following a 7-year delay following multi-modality treatment of a Ewing Sarcoma that included pre-operative chemotherapy and post-operative radiotherapy.

A 43 year-old man presented 7 years after treatment for a large Ewing’s sarcoma of the right mandible. The patient was treated with pre-operative chemotherapy, resection of the right hemi-mandible and had post-operative radiotherapy. This defect was not reconstructed primarily and the patient had considerable scar contracture over the defect, recessed chin and partial Andy-Gump deformity (Fig. 1a). Imaging revealed a defect that extended to the left mandibular body (Fig. 1b) and a remnant proximal right mandible, which included the right condylar head and neck. Additionally the remnant was rotated anteriorly (Fig. 1c). The patient had no cranial neuropathies, with intact function of both the marginal mandibular division of cranial nerve VII, and the mandibular division of cranial nerve V. With the inability to pre-bend a reconstruction bar, the considerable distortion of the remnant right condyle and the extent of the defect, this case underwent virtual surgical planning with a planned fibular free-flap for reconstruction (Materialize, USA).

A CT with 1 mm fine-cuts was used for pre-operative virtual planning. Figure 2 demonstrates the planned osteotomies for the resection and reconstruction. The defect extended from the right condylar neck to the left mandibular body. On the right, an osteotomy through the mandibular notch was designed. A 4-segment bony reconstruction was planned with three osteotomies, using the left leg, to restore mandibular continuity (Fig. 2b). Following the virtual planning session, anatomic resin models (pre and post-operative) and cutting guides were produced (Materialize, USA). A pre-bent 2-0 locking reconstruction bar was also supplied (Synthes USA).

The mandibular cutting guide was then used to prepare the remnant right condylar neck. The left fibula was harvested with a small skin paddle. Cutting guides were affixed to the fibula and following osteotomies the segment was attached to the pre-bent reconstruction bar (Fig. 3). Following inset (Fig. 3c), the peroneal artery was revascularized using standard microsurgical techniques to the facial artery. The peroneal vein was coupled to the external jugular vein using a vein coupler (Synovis, USA). Surgical outcomes can be seen in Fig. 3d. No secondary revisions were required and this patient had restoration of his anterior mandibular projection as well as improved mastication. This case required 3D VSP to achieve the satisfactory result. In particular the virtual planning allowed for appropriate repositioning of the right remnant segment in the mandibular fossa as well as restore anterior projection of the reconstructed mandible.

### Objective outcomes with VSP

While there are many potential advantages to using 3D planning for reconstruction of osseous defects, these benefits have only recently begun to be reported in an objective fashion. Table 1 summarizes the available literature with respect to precision/structural outcomes, operative times, post-operative function, cost and inclusion of a comparison group [10–12, 14, 26–34].

**Table 1:** Objective outcomes reported in 3D computer planning for osseous reconstruction of the mandible

Reference	Year	Patients (total)	Precision/ Structural Outcomes	Operative Time	Functional Outcomes	Cost Analysis	Comparison Group
Weitz et al. [26]	2016	50	Pre and Post-operative mean distance from angle to midline; Frequency of bony consolidation	Mean time from anastomosis to end of operation	NR	NR	26 patients without VSP
Monaco et al. [27]	2016	76	NR	Mean Operative Time	Post Operative Diet	NR	Comparison amongst three different phases – All virtually planned
Toto et al. [10]	2015	57	NR	Mean Operative Time	NR	Cost Analysis	All patients had stereolithographic models; 25 had prebent plates and cutting guides
Zweifel et al. [11]	2015	20	NR	Mean Reconstructive Time	NR	Cost Analysis	11 patients without VSP
Succo et al. [14]	2014	5	Pre & Post Operative Mean Differences	Mean Ischemia Time	NR	NR	Pre-operative plan and Post-operative imaging – All virtually planned
Avraham et al. [28]	2014	52	NR	Mean Operative Time	Dental Rehabilitation	NR	9 patients without VSP
Hanasono et al. [29]	2013	38	Pre & Post Operative Mean Change In Bony Landmarks	Mean Operative Time	NR	NR	Pre-operative plan and post-operative imaging – All virtually planned; 183 matched patients without VSP
Seruya et al. [30]	2013	68	NR	Mean Ischemia Time Mean Operative Time	NR	NR	58 patients without VSP
Wang et al. [31]	2013	10	Pre & Post Operative Mean Angle & Height Differences	NR	NR	NR	All virtually planned
Foley et al. [32]	2012	8	Pre & Post Operative Mean Differences In Anterior - Posterior & Transverse Dimension	NR	NR	NR	All virtually planned
Ciocca et al. [12]	2012	1	Pre & Post Operative Displacement of Mandible	Reconstructive Time	NR	Cost Analysis	Pre-operative plan and post-operative imaging
Sink et al. [33]	2012	8	NR	Mean Ischemia Time	NR	NR	None
Roser et al. [34]	2010	19	Pre & Post Operative Mean Distances & Volumes	NR	NR	NR	Pre-operative plan and post-operative imaging

*NR* not reported

There is objective data to suggest that VSP results in improved surgical precision [14, 29, 32, 34]. Execution of planned osteotomies is very accurate. Succo et al. reported the average difference between planned and executed osteotomies to be less than 1 mm (0.98 ± 0.77 mm) [14]. This translates to high accuracy with bony landmarks. Hanasono et al. evaluated the pre-operative and post-operative positions of the mandibular condyles, gonions and gnathion. They noted a change of 4.11 ± 3.09 mm with VSP as opposed to 6.92 ± 5.64 mm using traditional reconstructive methods [29]. Surgical precision was also well demonstrated by Foley et al., who examined the average surgical error in the anterior-posterior (AP) dimension as well as the transverse dimension for a series of 8 patients reconstructed with either iliac crest bone grafts (ICBG) or fibular free flap [32]. In this series the mean difference in the AP dimension was 0.2 mm for the ICBG and 0.9 mm for the free fibula flap. Furthermore the mean difference in the intercondylar and intergonial angle dimensions were 1.6 mm and 1.7 mm for the ICBG and 2.7 mm and 2.5 mm for the free fibula flap respectively. Accurate osteotomies do not necessary correspond to precision in plating with conventionally bent plates. Roser et al. compared the planned plate and final outcome with a mean plate overlap of only 59%. Accuracy does not appear to be sacrificed when double barrel vascularized flaps are utilized. In a case series of ten patients the mean neo-mandible angles were 124.29° ± 5.08° pre-operatively, compared to 123.88° ± 5.88° post-operatively. Furthermore vertical heights were 26.72 ± 1.44 mm pre-operatively and 27.04 ± 1.50 mm postoperatively respectively [31]. Finally VSP has been suggested to improve bony contact and overlap [35]. Recent data from Weitz et al. supports this concept by finding better rates of bony consolidation in the planned cases (84% vs. 62%) [26].

There is also evidence to suggest that using 3D planning results in improved operative times [11, 14, 24, 29, 30]. Time improvements can be attributed to several stages of the procedure. By using prefabricated cutting guides there is time gained during both the fibular osteotomy and contouring. Additionally, by using a pre bent reconstruction plate, time typically spent contouring is eliminated. This can shorten the time for both the fibular flap inset and plating. In the case example illustrated above, the osteotomy and contouring took approximately 40 min. Time for reconstruction (defined as time from osteotomies to fixation of reconstruction plate) has been reported to be an hour less with a mean time of 20.8 min in those pre–planned, compared to 88.2 min with freehand osteotomies [11]. Similarly, ischemia time has been reported to be decreased from 105 ± 29 mins to 75 ± 8 mins [14]. Two studies reported a total operative time savings of nearly 2–3 h with VSP when compared to traditional techniques [28, 29].

Two groups have investigated whether a reduced operative time, using virtual-assisted techniques, translated into a cost benefit. Zweifel et al. compared 9 cases using a fibula to 11 freehand osteotomies and reported a cost reduction of nearly $4,000 with either a prebent or milled plate [11]. In a larger study of 57 patients, three groups were compared in terms of mean operative time and cost [10]. The first group underwent back table osteotomies, the second in-situ osteotomies and the third pre-planned osteotomies with pre-fabricated cutting guides. The mean operating time was 707, 660 and 534 min respectively. This was associated with reduction in the total costs (incorporating both operating room and manufacturing costs) with the first group having a cost of $24,532.50, the second $23,202.60 and the third $20,950.48 [10]. While there are only a few cost benefit analyses currently, it would be reasonable to suggest that as this technology is more widely adopted the costs of production will decrease.

### Limitations of VSP

As with any evolving surgical technique there are disadvantages for VSP. Firstly, the planning for such procedures can take a number of weeks. In particular the phases for using 3D reconstruction include 1) a planning phase 2) a modeling phase 3) a surgical phase, including both the ablative and reconstructive steps, and now more recently 4) post-operative analysis [7, 14]. In the experience of Succo et al. the average time for completion of the planning and modeling phase was 15 ± 3 days [14]. In our experience a planning session can usually be completed in 45 min – 1 h. Obtaining surgical models and cutting guides typically requires 10–14 days. While this planning time may not be an issue in craniofacial surgery, benign head and neck tumors or ORN, it is an important consideration when managing aggressive malignancies. In these cases, total treatment package time, including surgery and post-operative adjuvant treatment, should not exceed 100 days, otherwise it may impact both tumor locoregional control and overall survival [36]. As VSP technology continues to evolve, the speed at which the pre-operative planning and templating is executed will also likely improve.

A second potential issue is an inability to adjust cutting guides if additional bone must be resected for oncologic reasons. This could be circumvented by the pre-operative computer planning of wider osteotomies and/or having several different pre-planned cutting guides in order to produce negative oncologic margins at the time of surgery [13]. The experience of Toto et al. did not find this to be a problem however and instead felt that pre–operative planning enabled them to better anticipate their oncologic resection margins and furthermore enabled an improved dialogue between the ablative and reconstructive surgeons [10].

Finally, despite the accumulating reports for the role of 3D osseous reconstruction, the literature is still limited in reporting objective outcomes. In a recent systematic review it was found that quantitative results were only measured in 30% of cases [37]. Two important points are illustrated in Table 1. Firstly when reported, objective measures are only partially reported, and do not cover the full spectrum of structural outcomes, operative time, functional outcomes and cost analysis. Secondly in 3D osseous mandibular reconstruction, the most under – reported results are the functional outcomes, which include mastication, swallowing, articulation and facial aesthetics. Mastication and diet achieved postoperatively were reported in the paper by Monaco et al., in which 62% of patients were able to tolerate a solid diet, an outcome largely facilitated by the use of dental implants in 60% of cases [27]. From their series, dental rehabilitation is more likely when patients have benign pathologies rather than malignant disease (96% vs 29%), a factor that also translates to placement of immediate implants (81% vs 0%). This is no doubt due to both post operative radiation and long term prognosis [27]. The importance of dental implantation in achieving functional outcomes was also reported by Avraham et al. whereby 63% of patient received dental implants, with 48% of these achieving functional dentition [28]. Furthermore they found that with VSP there was no incidence of implant malposition reducing the need for additional surgery. Neither of these reports provided objective measures such as measurements of dental occlusion [27, 28, 37]. Similarly while there is occasional mention of facial symmetry in the literature it is reported in a subjective fashion rather than an objective validated fashion [24, 37]. Increasing adoption of this technology will likely require reporting outcome measures in a standardized, objective and validated fashion. Currently none of the companies offering VSP include post-operative analysis as part of their service package. These limitations are likely contributors to the limited adaptation of VSP technology.

### The future of VSP

There are a number of recent technological and methodologic advancements that continue to refine the use of VSP for osseous reconstruction. Innovative methods for osteointegrated implant design have been used to perform complete dental rehabilitation in one operation [20]. Additionally, improvements in plate manufacturing now allows for custom bent plates with predetermined holes that can be patient specific. Finally, there are some innovative efforts being developed to include soft tissue and vessels in the virtual surgical plan. A haptics-assisted surgical planning program incorporates CT-angiography data to identify perforator location, projected pedicle length and orientation of the planned skin paddle [38].

## Conclusions

VSP for reconstruction of osseous defects is an evolving technology that currently offers the potential of accurate reconstruction and may save operative time and cost. In order to ensure objective measures are reported in a standardized fashion it would be beneficial for a 3D reconstructive working committee to be created in order to establish the guidelines needed to be followed in describing outcomes. At the very minimum it would be suggested that there be greater reporting of systematic objective outcomes with respect to structural results, operative times, functional analysis and costs, all of which would go a long way towards increased acceptance. Furthermore the analysis of outcomes would no doubt be better facilitated by a compulsory post operative analysis offered by the reconstructive modeling companies in addition to the pre – operative planning services they already provide.

## Abbreviations

3D: Three-dimensional
AP: Anterior-posterior
CAD: Computer-aided design
CAM: Computer assisted modeling
ICBG: Iliac crest bone graft
NR: Not reported
ORN: Osteoradionecrosis
SSO: Saggital split osteotomy
TMJ: Temporomandibular joint
VSP: Virtual surgical planning
